# Stitched vision transformer for age-related macular degeneration detection using retinal optical coherence tomography images

**DOI:** 10.1371/journal.pone.0304943

**Published:** 2024-06-05

**Authors:** Mohammad Mahdi Azizi, Setareh Abhari, Hedieh Sajedi

**Affiliations:** Department of Mathematics, Statistics and Computer Science, College of Science, University of Tehran, Tehran, Iran; Mayo Clinic Minnesota, UNITED STATES

## Abstract

Age-related macular degeneration (AMD) is an eye disease that leads to the deterioration of the central vision area of the eye and can gradually result in vision loss in elderly individuals. Early identification of this disease can significantly impact patient treatment outcomes. Furthermore, given the increasing elderly population globally, the importance of automated methods for rapidly monitoring at-risk individuals and accurately diagnosing AMD is growing daily. One standard method for diagnosing AMD is using optical coherence tomography (OCT) images as a non-invasive imaging technology. In recent years, numerous deep neural networks have been proposed for the classification of OCT images. Utilizing pre-trained neural networks can speed up model deployment in related tasks without compromising accuracy. However, most previous methods overlook the feasibility of leveraging pre-existing trained networks to search for an optimal architecture for AMD staging on a new target dataset. In this study, our objective was to achieve an optimal architecture in the efficiency-accuracy trade-off for classifying retinal OCT images. To this end, we employed pre-trained medical vision transformer (MedViT) models. MedViT combines convolutional and transformer neural networks, explicitly designed for medical image classification. Our approach involved pre-training two distinct MedViT models on a source dataset with labels identical to those in the target dataset. This pre-training was conducted in a supervised manner. Subsequently, we evaluated the performance of the pre-trained MedViT models for classifying retinal OCT images from the target Noor Eye Hospital (NEH) dataset into the normal, drusen, and choroidal neovascularization (CNV) classes in zero-shot settings and through five-fold cross-validation. Then, we proposed a stitching approach to search for an optimal model from two MedViT family models. The proposed stitching method is an efficient architecture search algorithm known as stitchable neural networks. Stitchable neural networks create a candidate model in search space for each pair of stitchable layers by inserting a linear layer between them. A pair of stitchable layers consists of layers, each selected from one input model. While stitchable neural networks had previously been tested on more extensive and general datasets, this study demonstrated that stitching networks could also be helpful in smaller medical datasets. The results of this approach indicate that when pre-trained models were available for OCT images from another dataset, it was possible to achieve a model in 100 epochs with an accuracy of over 94.9% in classifying images from the NEH dataset. The results of this study demonstrate the efficacy of stitchable neural networks as a fine-tuning method for OCT image classification. This approach not only leads to higher accuracy but also considers architecture optimization at a reasonable computational cost.

## Introduction

**Fig 1:**
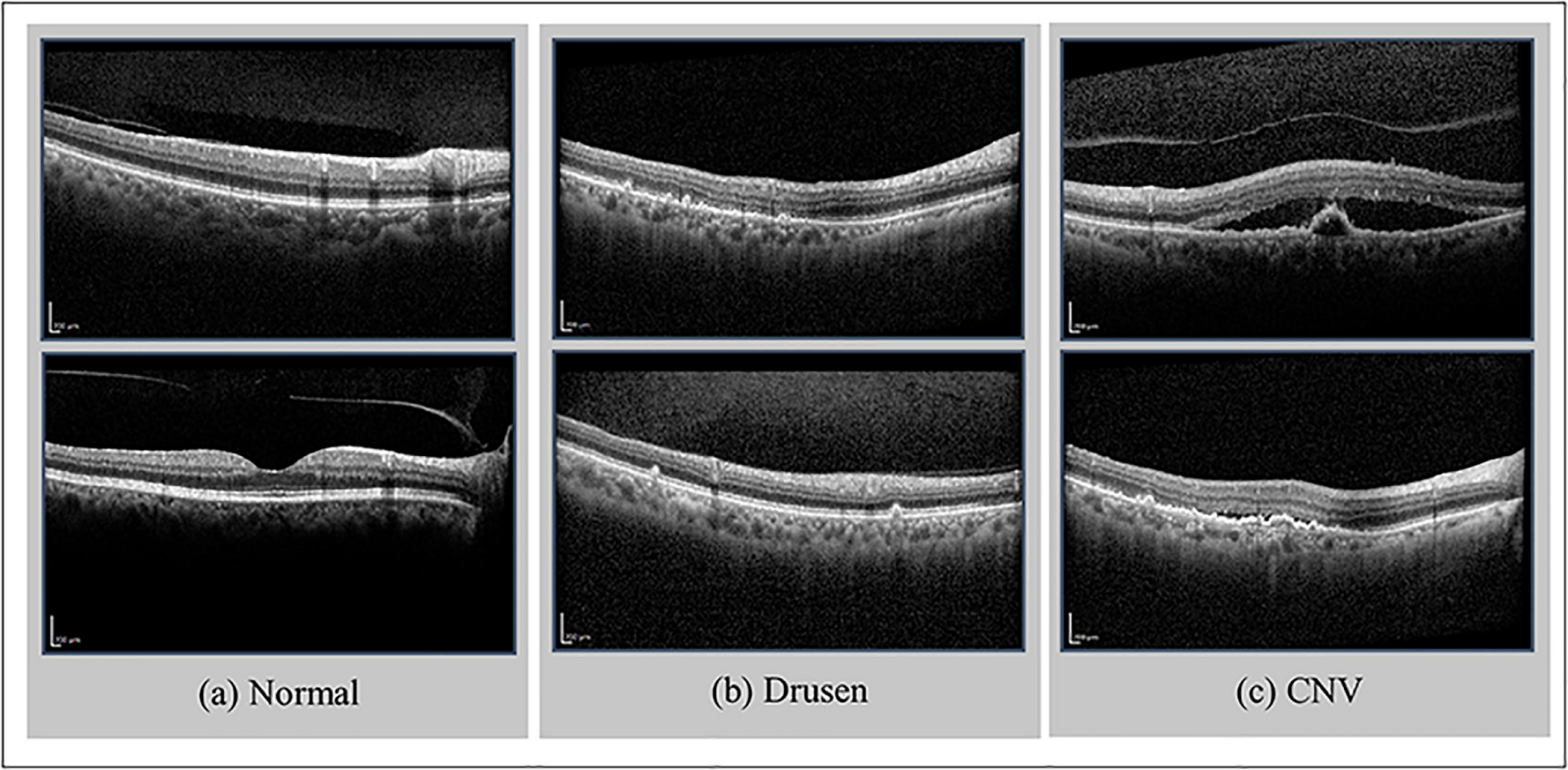
Sample of OCT retina images. Each column presents a pair of OCT retina images associated with a distinct label. The columns show classes labeled as (a) normal, (b) drusen, and (c) CNV.

Age-related macular degeneration is a retina-related disease that can lead to severe visual loss in patients aged 55 or above. Considering the increasing trend of the elderly population, estimates show that the number of people with AMD will rise from 196 million people in 2020 to around 288 million by 2040 [1]. AMD comes in two main types: dry and wet. The majority of AMD patients, about 80%, have the dry form, which typically results in minor vision loss that worsens slowly over time. However, the condition deteriorates more quickly for 10–15% of these patients, leading to geographic atrophy (GA). The key feature of dry AMD is the occurrence of drusen, yellowish deposits beneath the retina that vary in size and shape. However, the presence of small, fine drusen alone, without other signs, such as changes in the retinal pigment epithelium (RPE) or atrophy, should not be classified as AMD. There can also be diverse changes in pigmentation within the RPE. More severe stages of AMD are marked by the atrophy of the RPE, leading to GA, which can happen whether drusen is present or not. Dry AMD can evolve into the more severe form, wet AMD. The defining characteristic of wet AMD is the presence of CNV, characterized by the rapid growth of abnormal blood vessels beneath the retina. These vessels leak blood and fluid, causing inflammation and scarring, worsening vision loss. Wet AMD is almost always accompanied by some signs of simultaneous or pre-existing dry AMD, such as drusen, GA, or abnormalities in the RPE [2]. One of the methods used for early diagnosis of AMD and monitoring people in this regard is optical coherence tomography [3]. Accordingly, in recent years, automatic methods have been proposed to classify these images for faster early detection of AMD. Fig 1 shows an example of OCT images with normal, drusen, and CNV labels. It is worth mentioning that the challenge of distinguishing between various classes within a dataset is sometimes different. Specifically, in the context of our study, due to the slight differences between the images from drusen and normal classes, it is expected that the discrimination between images from these two classes could be more complex than the discrimination in the other two cases.

Automatic AMD detection using OCT images is an image classification task, which is a fundamental task in the realm of machine learning. Many of the primary methods for image classification were composed of a feature extraction module and a classifier head, such as a support vector machine (SVM), decision tree, and random forest. In such methods, the feature extraction module was a composition of different task-specific, well-curated image processing filters and techniques applied to images to get a vector representation for each image [4–8]. However, these methods, known as feature-based methods, could be computationally efficient and interpretable, but they lacked generalization accuracy. Also, they were unlikely to be able to leverage knowledge transfer and fast deployment for similar target datasets. With advancements in hardware and learning algorithms, it became possible to deploy deep neural networks. Convolutional neural networks (CNN), a specific kind of deep neural network, could automatically and adaptively learn spatial hierarchies of features from the training images. AlexNet [9], residual neural networks (ResNet) [10], and visual geometry group (VGG) [11] are well-known CNNs, utilized in a wide range of applications including retinal disease detection [12–22]. Also, vision transformers (ViTs) [23] are another category of state-of-the-art neural networks for computer vision tasks. However, while ViTs could achieve superior performance in large, well-known general datasets, because of the high data requirements in these models, they could not surpass the performance of CNNs in smaller medical datasets [24]. MedViT [25] is a model with a hybrid structure consisting of attention and convolutional modules, which was developed by Manzari et al. for robust classification of medical images and shown to provide better accuracy in the tested datasets than other known CNNs and transformers. Despite the success of this model, it is relatively large. It consists of multiple heterogeneous stages and layers, which require a curated approach to transfer learned knowledge and to use it as a backbone for architecture search to achieve an optimal model in an accuracy-efficiency trade-off on a new, similar, and even smaller dataset.

Pan et al. introduced neural networks stitching [26] for environmentally friendly artificial intelligence (Green AI) [27] purposes as an architecture search method for fast deployment of an optimal model in terms of accuracy-efficiency trade-off using multiple pre-trained models, referred to as anchors. Despite the aim of proposing stitchable neural networks, it is not clearly explained how the performance of the method is in more practical scenarios in which the target dataset is out-of-distribution of the pre-training (source) dataset. To elucidate the complete picture of the performance of stitchable neural networks, we must compare the results of the zero-shot scenario (where the model is just trained on the source dataset and directly tested on the whole of the target dataset), the out-of-distribution stitching scenario (where the anchors are pre-trained on source datasets but the stitching phase is performed on the target dataset in a limited number of epochs and finally the candidate models are evaluated on the target dataset), and the in-distribution stitching scenario (where the target and source dataset are the same and the results of this scenario provide an upper bound of performance in a constrained number of epochs).

This study aims to address several knowledge gaps in the field of OCT image classification using stitchable neural networks. These gaps can be summarized as follows:

- Previous studies have not thoroughly investigated the performance of stitchable neural networks as a fine-tuning method for domain-specific or medical image classification tasks.
- The performance gap of stitchable neural networks between out-of-distribution and in-distribution scenarios remains unclear in the existing literature.
- The performance of the proposed backbone architecture in zero-shot classification requires further investigation.

In this study, we tried to solve the classification problem of the second version of the NEH dataset [28], considered a small dataset, with the help of stitching pre-trained MedViT models. Our study aimed to accomplish the following objectives: 1) Introduce a zero-shot test setup for OCT image classification using publicly available datasets and establish MedViT models as baselines for future research., and 2) Demonstrate the effectiveness of stitchable neural networks as a fine-tuning method, considering both model weights and architectural optimization in both in-distribution and out-of-distribution scenarios within the domain of OCT image classification. We have considered the following two cases to stitch MedViT models:

1. Anchors were pre-trained on the University of California San Diego (UCSD) dataset [29] (out-of-distribution stitching).
2. Anchors were pre-trained on the NEH dataset, and as a result, the pre-training dataset and the target were the same (in-distribution stitching).

The principal difficulty in training the MedViT model on the NEH dataset is that this dataset is imbalanced and can lead to models that are inherently biased toward the majority class, resulting in poor performance on the minority class, which is often the class of interest. Focal loss [30], an extension of cross-entropy loss, was proposed to address the challenges associated with training on imbalanced datasets. It achieves this by assigning greater importance to the loss derived from more complex samples. Considering the high number of parameters of the MedViT family models, we introduced a smaller model of this family called micro MedViT, and we have shown that despite the lower number of parameters, this model obtained competitive results compared to the tiny MedViT model. Next, we stitched together two different models of trained MedViTs to improve the results. The main challenge for stitching MedViT models is the heterogeneity of layers in each stage and the high number of layers in the third stage, which increases the number of candidate models in the search space. The main contribution of this article can be summarized as follows:

1. We have introduced the micro model from the MedViT family and demonstrated its results, which were comparable to those of the tiny model despite the smaller number of parameters.
2. We have evaluated the stitching method’s results in a more realistic case, in which the pre-training dataset differs from the stitching dataset.
3. Finally, we have shown the promising performance of the proposed method on the small NEH dataset.

## Related works

This section briefly reviews the related works on automated AMD detection based on retinal OCT images.

### Feature-based methods

In some studies [4–8], the features of OCT images are extracted after applying multiple image processing filters. Then, images containing signs of AMD are recognized with the help of the extracted features and a traditional classifier head. In particular, [4] uses features based on local histograms and a Bayesian network classifier. Srinivasan et al. recognized the images labeled as normal, AMD, and diabetic macular edema (DME) with 86.67, 100, and 100% accuracies by using an SVM classifier on the features obtained from the histogram of oriented gradients (HOG) descriptors [5]. Sun et al. also separated spectral domain OCT (SD-OCT) images with normal, AMD, and DME labels based on dictionary learning and three SVM classifiers [6]. In [7], an algorithm based on dictionary learning and random forest classification is presented, and it is shown that this method performs similarly to humans on European genetic database OCT images. In 2023, Liew et al. introduced a manual method called multi-sized kernel echo-weighted median patterns (MSKξMP), which is a generalized version of the local binary patterns (LBP) method, to extract the features of OCT images and these features are used to classify OCT images with classifiers such as adaptive boosting (AdaBoost), naïve Bayes, SVM, random forest and random undersampling/boosting (RUSBoost). This research has shown that for different datasets of OCT images, the SVM classifier with polynomial kernels performs better than other classifiers [8].

### Deep learning-based methods

To comprehensively represent the literature on deep learning-based methods, we have categorized related studies into three subgroups: traditional CNNs, multi-scale CNNs, and vision transformers.

#### Traditional CNNs

Due to the power of deep learning-based models in automatic feature extraction, these models have been used in recent years to improve classification results on larger datasets. In [12,13], modified VGG and AlexNet models were used to classify OCT images. Also, Kermany et al. in [14] and Li et al. in [15], with the help of transfer learning and using the pre-trained Inception-v3 and 16-layer VGG (VGG16) networks on the UCSD dataset, obtained 96.6% and 98.6% accuracies, respectively. Serener et al. showed that the 50-layer ResNet (ResNet50) performs better than AlexNet for detecting images with wet AMD and dry AMD labels [16]. In [17], ResNet50, Inception-v3, and VGG16 networks were trained based on transfer learning to recognize images labeled normal, wet AMD, and dry AMD, and it was shown that the Inception-v3 network performed better than the other two networks.

In 2023, Han et al. have used VGG16, VGG19, and ResNet50 as image feature extractors in the transfer learning method and showed that VGG19 performs better than the other two networks in classifying OCT images for neovascular age-related macular degeneration (nAMD) and central serous chorioretinopathy (CSC) diagnosis. In this study, a private OCT dataset was collected and utilized. The nAMD class consists of polypoidal choroidal vasculopathy (PCV), retinal angiomatous proliferation (RAP), and typical nAMD cases. The CSC class also consists of chronic and acute CSCs. In addition, mix-up data augmentation, a data augmentation technique that generates mixed samples and labels with weighted averaging of multiple samples and their suitable labels from the training set, was used to improve the robustness and accuracy of the presented model [18]. In [31], a method based on adversarial learning is presented for training a deep network that can extract robust features against domain shift with the help of two datasets of retinal OCT images and another auxiliary network to classify images into normal and abnormal classes. In [19], two ResNet50 models were used for the two-stage classification of OCT retinal images into four normal categories and three phases of dry AMD, including atrophy-associated drusen regression, GA, and nascent GA (nGA). In the first step of the presented structure, the images are placed in three categories: normal, drusen & nGA and GA, and in the second step, the images containing drusen and nGA tags are separated. In [20], a modified VGG19 network has been used to classify OCT and optical coherence tomography angiography (OCTA) images to diagnose four eye diseases. In [32], the interpretable network modeled with the reciprocal learning (InterNRL) framework is introduced to achieve a model with interpretability and high accuracy based on the student-teacher structure and benefit from an interpretable student model and an accurate teacher model. The final model obtained in this study for the second version of the NEH dataset has achieved 91.9% accuracy. In a comparative study [33], Aykat et al. investigated the performance of five different models, including the 201-layer densely connected convolutional network (DenseNet121), enhanced small, efficient convolutional neural network (EfficientNet-v2-s), convolutional neural networks for mobile vision applications (MobileNet), extreme Inception (Xception), and Inception-v3. Results of this study show that DenseNet121 outperforms other models in classification accuracy on the 8-class OCT dataset (OCT-C8), which is comprised of images with eight labels: central serous retinopathy (CSR), diabetic retinopathy (DR), macular hole (MH), AMD, CNV, DME, drusen, and normal. The knowledge-driven fine-grained wet AMD classification (KFWC) model is introduced in [34]. KFWC design is inspired by how medical professionals diagnose diseases based on recognizable lesion signs from color fundus photography (CFP) and OCT images. To this end, KFWC utilizes a split-attention network (ResNeSt) [@] backbone in the pre-training phase for multi-label lesion sign acquisition and then, in the fine-tuning phase, leverages bi-modal feature fusion for better accuracy in multi-class wet AMD classification. In [21], a hyperparameter fine-tuning investigation was conducted on four popular CNN-based models, including AlexNet, VGG, Inception, and ResNet, to achieve the optimum training configuration of the models in the context of retinal disease detection using OCT images. Choudhary et al. employed transfer learning on a VGG19 with customized top-notch layers to classify OCT images into AMD, DME, drusen, and normal classes and achieved superior classification accuracy compared to ResNet50 and Inception-v3 models [22]. In [35], a novel self-distillation method called noise regularized self-distillation (NRSD) was introduced to achieve generalizable models based on ResNet18, MobileNet-v2, and ShuffleNet-v2 for OCT image classification from the UCSD dataset. Label smoothing is a widely utilized machine learning technique that prevents overfitting by perturbing labels during training. Kaloi et al. proposed a novel weighted label smoothing loss regularization (WLSLR) and applied it to a CNN-based multi-task dual-stream model for retinopathy classification using CFP and OCT images [36].

#### Multi-scale CNNs

In [37], Rasti et al. were able to classify the three classes of normal, AMD, and DME in the NEH dataset with a score of 0.9985 in the area under the receiver operating characteristic curve (AUC) criterion by assembling multi-scale CNNs and using a new cost function. Similar to [37,38] also combines features obtained from the multi-scale CNN structure. However, unlike the method proposed by Rasti et al., which needed to tune the loss function, a cost-sensitive loss function that does not need to be tuned has been used, leading to better modeling of the imbalanced UCSD dataset. In [39], the iterative fusion convolutional neural network (IFCNN) model, which iteratively fuses the features of each layer with all the previous layers in order to take advantage of the low and high-level features in CNN networks, was used for the classification of UCSD data images. Also, Thomas et al. extracted the local structures of the images by using filters with different sizes in the multi-scale CNN structure and obtained a weighted average accuracy of 99.73% for the binary classification of normal vs. AMD in UCSD data [40]. In 2022, Sotoudeh-Paima et al., while introducing the second version of the NEH dataset, were able to achieve 93.4% accuracy on the second version of the NEH dataset with the help of a multi-scale structure to combine the features of a pre-trained VGG16 model on UCSD and ImageNet data. Since the volume of NEH was smaller than UCSD, using the pre-trained model on UCSD and a large general image database, ImageNet, led to a 1.2% increase in the average accuracy of the model compared to using the pre-trained model on only the ImageNet dataset [41]. Even though fusing multi-scale features allows the model to use both high and low-level features, the time and cost of model learning and inference increases compared to classical CNN models due to several different CNN structures, from shallow to deep. In [42], using a scale-adaptive network, multi-scale features were extracted from the input images, and further, with the help of fusing the extracted features in a hierarchical structure, the classification accuracy on UCSD data was improved to 99.69% in the five-fold cross-validation method. Their multi-task model exhibited competitive results relative to previous state-of-the-art models. In recent work, Baharlouei et al. presented a wavelet scattering transform (WST)-based architecture to capture fine to coarse, robust features from OCT images. They used a principal component analysis (PAC) classifier on top of two WST layers. They showed that, despite the low computational complexity of the model, the proposed model could achieve state-of-the-art results [43]. In [44], Almasganj et al. investigated three feature pyramid networks, named pruned feature pyramid network, bi-directional feature pyramid network (Bi-FPN), and path aggregation network (PA), for OCT classification. They have shown using these feature pyramid networks with a VGG16 pre-trained backbone could achieve an accuracy of 94.6%, 94.8%, and 95% in five-fold cross-validation on NEH ternary classification.

#### Vision transformers

In recent years, after the introduction of ViTs, different versions of vision transformers could perform better in many computer vision tasks than CNNs on datasets in different domains. Despite this issue, insufficient studies have been conducted to apply transformers on OCT images to diagnose AMD. In 2023, a comparative study conducted by Zhou et al. it was demonstrated that ViT has superior performance over VGG16, ResNet50, and EfficientNet [45]. In [46], a technique termed model-based transformer (MBT) based on feature extraction after fine-tuning pre-trained ViT and the shifted window (Swin) transformer models are presented to increase the accuracy of OCT image classification. He et al. [47] utilized the Swin transformer architecture along with a loss function called Polyloss to improve the classification results of OCT images for retinal disease detection. Polyloss is calculated by perturbing the first coefficient of Taylor expansion of cross-entropy loss and is designed to improve classification results of neural networks in different domains. In [48], a ViT-based masked autoencoder was employed in a self-supervised manner to learn robust representations for OCT and CFP images by reconstructing 1.6 million retinal images from their masked versions. Table 1 provides a brief review of deep learning-based methods for retinal disease detection using OCT images in 2023.

**Table 1:** A brief review of deep learning-based methods and their results in 2023.

Study	Category	Method\Model	Data	Classes	Results
Han et al. [18]	Traditional CNNs	VGG16, VGG19, and ResNet	A local OCT dataset	Normal, nAMD (PCV, RAP, and typical), and CSC (acute and chronic)	3-class best accuracy: 99.7%, and 6-class best accuracy: 91.1%
Wang J et al. [31]	Traditional CNNs	Domain adaptation-based learning	A local OCT dataset	Normal, and abnormal (abnormal class is subtyped to 4 classes based on the location of the lesion)	5-class accuracy: 94.52%
Hu et al. [19]	Traditional CNNs	Hierarchical classification based on ResNet50 and image enhancement	A local OCT dataset	Normal, drusen, nGA, and GA	Macro-f1: 91.32%, and Kappa: 96.99%
Udayaraju et al. [20]	Traditional CNNs	Multilayered classification model with VGG-19	UCSD	Normal, drusen, CNV, and DME	Accuracy: 99.45%, and f1-score: 98.56%
Wang C et al. [32]	Traditional CNNs	Prototype-based classifier (ProtoPNet), and global image classifier (GlobalNet), and proposed InterNRL framework	NEH-v2	Normal, drusen, and CNV	Accuracy: 91.9%, and sensitivity: 91.0%
AYKAT et al. [33]	Traditional CNNs	DenseNet121, hyperparameter-tuning	OCT-C8	Normal, drusen, CSR, AMD, CNV, DME, DR, and MH	Accuracy: 97.79%, and sensitivity: 97.69%
Haihong et al. [34]	Traditional CNNs	KFWC, ResNeSt50, pre-training	A bi-modal local dataset	PCV, nAMD, and others	Kappa: 0.8507, and f1-score: 0.9247
Stanojević et al. [21]	Traditional CNNs	Inception, hyperparameter-tuning	UCSD	Normal, drusen, CNV, and DME	Accuracy: 0.9553, and f1-score: 0.93687
Choudhary et al. [22]	Traditional CNNs	VGG19, transfer learning	UCSD	Normal, drusen, CNV, and DME	Accuracy: 99.17%, and sensitivity: 99.00%
Paluru et al. [35]	Traditional CNNs	NRSD framework, ResNet18, MobileNetV2, and ShuffleNetV2	UCSD, NEH-v1, and Duke	Normal, drusen, CNV, and DME (UCSD); Normal, AMD, and DME (Duke and NEH-v1)	UCSD best accuracy: 0.95, NEH-v1 best accuracy: 0.91, and Duke best accuracy: 0.84
Kaloi et al. [36]	Traditional CNNs	WLSLR, multitasking, dual-stream CNN	UCSD	Normal, drusen, CNV, and DME	Accuracy: 99.8%
Akinniyi et al. [42]	Multi-scale CNNs	Multistage classification, multi-scale feature fusion	UCSD	Normal, drusen, CNV, and DME	Five-fold accuracy: 94.26%
Baharlouei et al. [43]	Multi-scale CNNs	WST, PCA	OCTID, TOPCON, Duke, and Heidelberg	Normal, AMD, and DME (Duke and Heidelberg); Normal, DME (TOPCON); Normal, AMD, CSR, DR, MH (OCTID)	Duke accuracy: 97.1%, Heidelberg accuracy: 94.4%, TOPCON accuracy: 96.6%, 2-class OCTID accuracy: 100%, and 5-class OCTID accuracy: 82.5%
Almasganj et al. [44]	Multi-scale CNNs	VGG16, feature pyramid networks	NEH-v2	Normal, drusen, and CNV	Accuracy: 95.0%, and f1-score of 0.925
Zhou Z et al. [45]	Vision transformers	ViT, and symmetric cross-entropy loss function	UCSD	Normal, drusen, CNV, and DME	Accuracy: 96.57%
Hammou et al. [46]	Vision transformers	ViT, Swin transformer, approximate sparse representation, and transfer learning	OCTID, and a local OCT dataset	Normal, AMD, CSR, DR, MH (OCTID); Normal, wet AMD, DME, ERM, and MH (local)	OCTID accuracy: 82.76%, and accuracy for the local dataset: 96.83%
He et al. [47]	Vision transformers	Swin transformer, Polyloss	UCSD, OCT-C8	Normal, drusen, CNV, and DME (UCSD); Normal, drusen, CSR, AMD, CNV, DME, DR, and MH (OCT-C8)	UCSD accuracy: 0.9980, and OCT-C8 accuracy: 0.9711
Zhou Y et al. [48]	Vision transformers	ViT, autoencoder, pre-training	Firstly, the proposed autoencoder model pre-trained on 1.6 million retinal images—collected from numerous public retinal datasets (OCT, CFP). Then it trained in a supervised manner on multiple datasets and achieved state-of-the-art performance in different tasks for ocular disease diagnosis and prognosis.		

### Hybrid and ensemble methods

Huang et al. were able to record better performance than the IFCNN network by using the features extracted by a segmentation network in the proposed model called Layer Guided CNN, which led to an increase in focus in more meaningful areas to detect damage caused to the retina [49]. Also, in [50] Fang et al., a new method called lesion-aware convolutional neural network is introduced, which uses a lesion detection network to create an attention map for weighting the information obtained from local convolutional filters, increasing the classification accuracy in exchange for increasing the computational complexity (due to the use of an independent network to produce the attention map). In 2020, Das et al. introduced B-scan attentive CNN, which uses the self-attention mechanism in the convolutional network structure and emphasizes the more important information of B-scan 3D OCT images to perform better classification [51]. In [52], the deep retinal analysis and grading framework (RAG-FW) was introduced and obtained an average accuracy of 98.70% for detecting the degree of retinal damage on several datasets of OCT images as a hybrid convolutional framework of classification and segmentation networks.

In [53], three different methods of using only one classifier, using a classifier and the segmentation output of the retinal layer and fluid segmentation network (RelayNet) model, and using a classifier and the segmentation output of the graph-cut method have been compared to classify OCT images in seven different classes and have shown the use of segmentation information leads to an increase in the accuracy of the final model. In [54], using an optimized algorithm for retina image segmentation and an ensemble structure of bagged trees and two deep learning models for 2D and 3D images, a classifier of SD-OCT images into non-, early, and intermediate AMD classes was introduced. In [55], the dual guidance network for the classification and segmentation of OCT images is presented. The dual guidance network uses information related to one task to improve the results of another task. This feature improves the results of both tasks, but on the other hand, due to the sequential nature of training and testing in these networks, the complexity of the network increases compared to multitask networks. Another limitation of these networks is the need for a dataset with class and pixel-wise labels simultaneously. The global attention block network (GABNet), which combines a global attention block and convolutional neural networks, was introduced for classifying OCT retina images [56]. In [57], the tri-branch CNN method is presented, which aims to provide a multi-modal classifier for better diagnosis of AMD, with the help of features extracted from OCT and CFP images and combining them based on the attention mechanism. Furthermore, in this study, to overcome the problem of lack of multi-modal data, a generative adversarial network (GAN) is designed and used. In [58], using the Inception ResNet-v2 model as an image feature extractor and combining classical classifiers, the classification process has been performed on the dataset of OCT images with five classes. Khan et al. in [59] employed a method based on the use of pre-trained models of DenseNet201, Inception-v3, and ResNet50 neural networks, in which after optimizing the features extracted with the help of neural networks, k-nearest neighbors (KNN) and SVM classifiers are ultimately used to determine the class of each item. In [60], the hybrid ensemble deep network (HEDN) model based on the MobileNet-v2, ResNet50, and VGG16 models is presented to classify OCT retinal pathology images into four classes. Furthermore, Maurya et al. in [61] managed to reach 99.8% accuracy on the test data of the UCSD dataset by fine-tuning the MobileNet-v1, EfficientNet-B3, network architecture search network for mobiles (NASNetMobile) and Xception models and assembling them. In [62], a speckle reduction filter was applied to OCT images. Then, the features of preprocessed images were extracted by an explainable custom fully dense convolutional neural network (FD-CNN) and fed to the proposed deep SVM and deep KNN classifiers. The proposed method achieved state-of-the-art results on the UCSD and Duke datasets. Celebi et al. in [63] preprocessed OCT images for region of interest (ROI) selection and noise reduction. They applied an optimized Bayesian non-local mean (OBNLM) filter for noise reduction on OCT images. Then, in the training phase, they utilized the capsule neural network (CapsNet) model for high-level feature extraction. In the proposed architecture in this study, extracted features from CapsNet were fed to a 3-layer fully connected neural network for classifying OCT images into four classes: normal, drusen, dry AMD, and wet AMD. Gan et al. [64] gathered an OCT dataset, including images with retinal-vein occlusion (RVO), CSC, AMD, and DME labels. They manually created ROI masks for each image and then employed them in cropping OCT images in a preprocessing step. The proposed method in this study leveraged Inception-v3, ResNet34, and VGG13 as feature extractors. In this method, all extracted features were fused in a PCA module. Next, the least absolute shrinkage and selection operator (Lasso) method is used for feature selection. Then, the selected features were input into a classifier. In this study, SVM exhibited superior performance in terms of accuracy and AUC compared to KNN, Extra Trees, logistic regression (LR), and multilayer perceptron (MLP). Dutta et al. used ResNet50, Inception-v3, along with ViT for feature extraction. Concatenating the outputs of ViT and CNNs, as done in this study, could capture both local and global features of input images. However, it leads to an increase in the dimensionality of the feature vectors. Furthermore, it does not address the issue of high data requirements, a characteristic commonly associated with purely attention-based models such as ViTs [65]. Table 2 briefly reviews the studies that utilized hybrid and ensemble methods in 2023.

**Table 2:** A brief review of hybrid and ensemble methods and their results in 2023.

Study	Method\Model	Data	Classes	Results
Kaothanthong et al. [53]	RelayNet segmentation, graph-cut segmentation, and ResNet50 classifier	APTOS-2021	Normal, nAMD, PCV, DME, RVO, CME, and others	Accuracy: 94.80%, and f1-score: 76.03%
Moradi et al. [54]	Graph-cut segmentation, bagged trees classifier, 2D and 3D deep neural networks	Zeiss, and Bioptigen	Non-AMD, and intermediate AMD (Bioptigen); None-AMD, early AMD, and intermediate AMD (Zeiss)	Zeiss accuracy: 96.67%, and Bioptigen accuracy: 89.16%
Diao et al. [55]	Dual guidance CNN	UCSD, and a combination of the RETOUCH and Duke datasets	Normal, drusen, CNV (UCSD); Normal, and macular edema (the combinatory dataset)	UCSD accuracy: 96.93%, and an accuracy of 97.00% on the combinatory dataset
Huang et al. [56]	Global attention block, Xception	UCSD	Normal, drusen, CNV, and DME	Accuracy: 0.99, and f1-score: 0.99
Wang Q et al. [57]	Generative adversarial network, multimodality	Peking	Normal, dry-AMD, wet-AMD, and PCV	Accuracy: 84.9%, and f1-score: 86.2%
Pin et al. [58]	Inception ResNet-v2, ensemble voting classifier	Soonchunhyang	AMD, DME, CSC, branch retinal vein occlusion, central retinal vein occlusion	Accuracy: 97.68%, and sensitivity: 97.42%
Khan et al. [59]	Ant colony optimization, Inception-v3, ResNet50, and DenseNet201	Soonchunhyang	AMD, DME, CSC, branch retinal vein occlusion, central retinal vein occlusion	Accuracy: 99.1%, and AUC: 1
Priya et al. [60]	Ensemble neural network, MobileNet-v2, ResNet50, and VGG16	UCSD	Normal, drusen, CNV, and DME	Accuracy 97.3%, and f1-score: 96.5%
Maurya et al. [61]	Guided-ensemble, MobileNet-v1, EfficientNet-B3, NASNetMobile and Xception	UCSD	Normal, drusen, CNV, and DME	Accuracy: 99.8%
Kayadibi et al. [62]	Deep SVM, fully dense fusion neural network	UCSD	Normal, drusen, CNV, and DME	Accuracy: 99.60%, f1-score: 99.60%
Celebi et al. [63]	ROI selection, CapsNet, OBNLM	UCSD, and a local dataset	Wet AMD, dry AMD, drusen, and normal	UCSD accuracy: 98.07%, and UCSD f1-score: 98.07%
Gan et al. [64]	Inception-v3, ResNet34, VGG13, PCA, Lasso, SVM	a local dataset	CSC, AMD, DME, RVO	Accuracy: 93.8%
Dutta et al. [65]	ResNet50, Inception-v3, ViT	UCSD, and OCTID	Normal, drusen, CNV, and DME (UCSD); Normal and AMD (OCTID)	UCSD accuracy: 94.46%, and UCSD f1-score: 0.94, OCTID accuracy: 92.37%, and OCTID f1-score: 0.92

In numerous studies in the realm of medical image classification, different CNNs have served as classifiers or feature extractors because of their advantage in automatic generalizable feature extraction of OCT images, surpassing traditional image processing-based feature extractors. Some of the reviewed studies have incorporated attention blocks into their proposed hybrid architectures as global feature selectors to improve the performance of traditional CNNs [56,65]. Global attention blocks [56] generate attention maps by utilizing multiple low-cost max and average pooling layers, and do not include the standard high performance self-attention. The CNN-transformer [65] architecture is an ensemble of the ViT, ResNet50, and Inception-v3 models and does not address the high-data requirement of ViT, particularly in non-transfer learning scenarios. MedViT is the backbone model of the proposed stitching method in this study. It comprises various hybrid blocks that are designed to harness the advantage of CNNs and transformers, long-range and short-term feature extraction, and overcome the high-data requirements of the ViTs. Furthermore, prior research either trained models from scratch or fine-tuned pre-existing general-purpose networks, neglecting the optimal model size for the target dataset. The findings of this study highlight the effectiveness of utilizing fine-tuning methods, such as stitchable neural networks, to achieve an optimal model in the efficiency-accuracy trade-off.

## Method

In this part, the architecture of MedViT is first reviewed as the base transformer for stitching. Afterward, the proposed method, which employs stitchable neural networks, is described.

### MedViT

MedViT is a hybrid structure consisting of convolutional and transformer blocks developed by Manzari et al. MedViT using multi-scale image information improves robustness and accuracy in medical image classification. In particular, MedViT consists of efficient convolutional blocks (ECBs) and local transformer blocks (LTBs). Although both ECB and LTB blocks use a hybrid structure, each focuses more on local and global patterns, respectively. In the following, we will briefly review each of the blocks.

#### Efficient convolutional block

**Fig 2:**
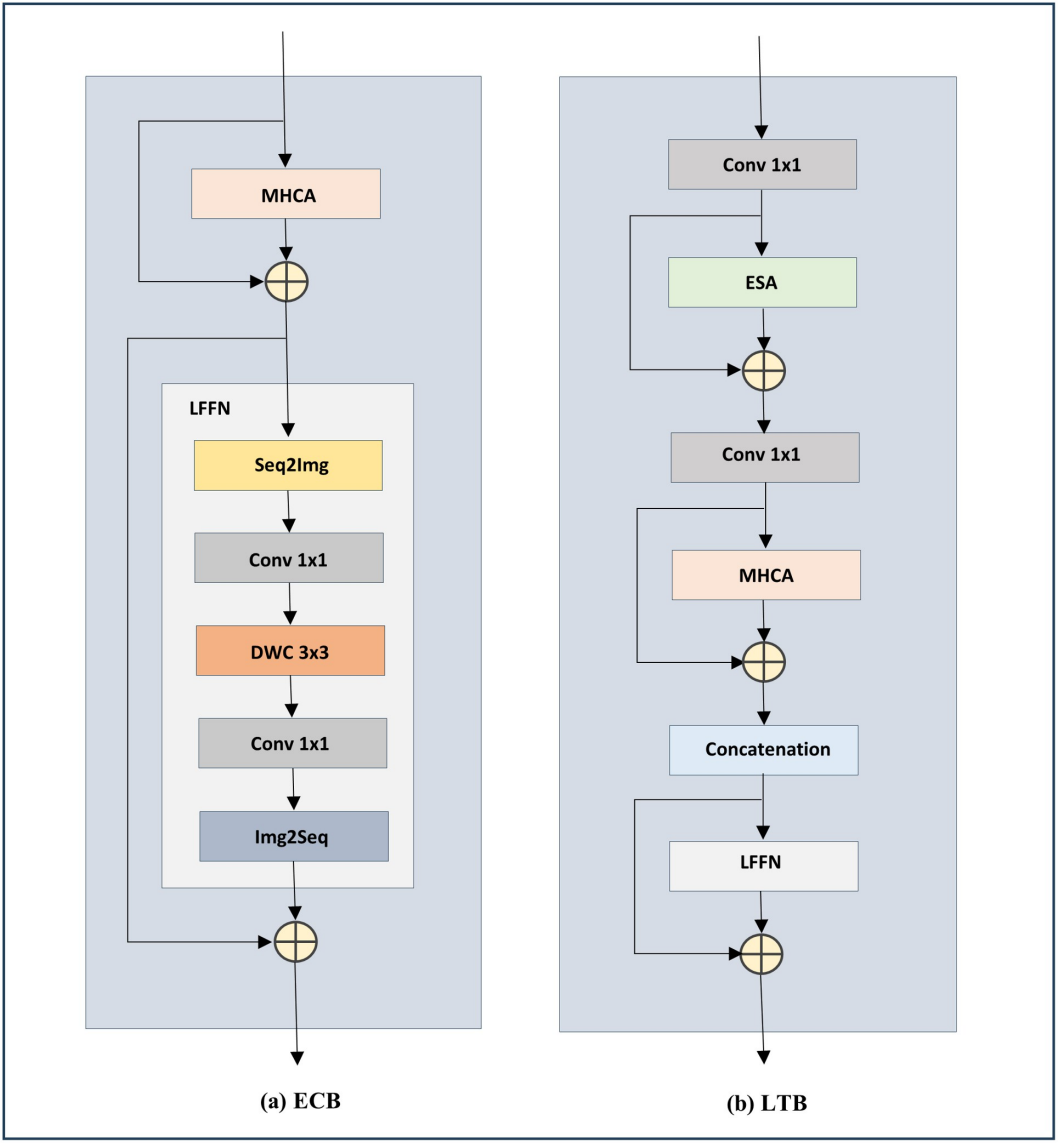
Structure of (a) ECB and (b) LTB blocks. Blocks ECB and LTB both comprise layers LFFN and MHCA. However, an ESA layer is present at the beginning of the LTB block. In the shown structure of the ECB block, the inner structure of LFFN is uncovered. ‘Conv 1x1’ and ‘DWC 3x3’ each denote a convolution with a kernel size of 1 and a depthwise convolution with a kernel size of 3, respectively. ‘Seq2Img’ is a function that reshapes a sequence of inputs to a two-dimensional output. Conversely, ‘Img2Seq’ serves as the inverse operation of ‘Seq2Img’.

ECB is an attention-based structure and consists of multi-head convolutional attention (MHCA) and locally feed-forward network (LFFN) layers. ECB is presented to better learn the complex medical tissues in the background of the images, increasing the model’s accuracy. According to (1), the MHCA layer is obtained from the inner product of a projection layer in the concatenation of convolutional attentions (CA) outputs. MHCA, as an attention layer, makes the model pay attention to the points containing more critical information, and the LFFN layer learns local structures using the depthwise convolution (DWC) sublayer. Fig 2a shows the structure of LFFN and ECB.


$$\begin{array}{c} MHCA\left(X\right)=Concat\left(CA_{1}\left(x_{1}\right),CA_{1}\left(x_{2}\right),\ldots ,\;CA_{h}\left(x_{h}\right)\right)W^{O}\\ CA\left(x\right)=\;W.T_{\left\{i,j\right\}}\;\;\;where\;\;\;T_{\left\{i,j\right\}}\in x \end{array}$$ (1)

Where *X* = [*x*_1_,*x*_2_,…,*x*_*h*_] is the input of the MHCA layer, and *h* indicates the number of heads in MHCA. Furthermore, *W* are learnable weights in CA, *T*_{*i*,*j*}_ represents adjacent tokens in the input, and *W*^*O*^ is the output projection layer.

#### Locally transformer block

LTB also follows the same structure as ECB, with the difference that an efficient self-attention (ESA) module has been added at the beginning to take advantage of the transformer in tracking global information. According to (2), ESA works similarly to multi-head self-attention, a standard structure in transformers. The noteworthy point in the ESA module is the use of the average pool in order to reduce the computational cost. Fig 2b shows the LTB structure.


$$\begin{array}{c} ESA\left(X\right)=Concat\left(SA_{1}\left(x_{1}\right),SA_{1}\left(x_{2}\right),\ldots ,\;SA_{h}\left(x_{h}\right)\right)W^{O}\\ SA\left(x\right)=\;Attention\left(x.W^{Q},P_{s}\left(x.W^{K}\right),P_{s}\left(x.W^{V}\right)\right) \end{array}$$ (2)

In the above relation, attention means standard scaled dot-production attention, and *P*_*s*_ means average pool operator with a stride s. Also, *W*^*Q*^, *W*^*K*^ and *W*^*V*^ are learnable attention weights.

#### Micro MedViT

The MedViT models introduced in [25] include tiny, small, and large models, each with 31.14, 44.41, and 57.68 million parameters, respectively. In this study, we added the micro MedViT model with 24.50 million parameters to the family of MedViT models, which provides the same performance as the tiny model despite the smaller number of parameters. The only difference between this and other models is the number of layers of the third stage and the amount of path dropout. The value of path dropout for the tiny model is equal to 0.1, and for the micro model, it is 0.05. Table 3 gives a complete specification of the architecture of tiny and micro models.

**Table 3:** Full architectural specifications of micro and tiny MedViTs.

Stages	Layer type	Tiny MedViT	Micro MedViT
Stem	Convolution layers	Conv 3 **×** 3, C = 64, S = 2	
		Conv 3 **×** 3, C = 32, S = 1	
		Conv 3 **×** 3, C = 64, S = 1	
		Conv 3 **×** 3, C = 64, S = 2	
Stage 1	Patch embedding	Conv 1 **×** 1, C = 96	
	MedViT block	[*ECB*^96^ × 3] × 1	
Stage 2	Patch embedding	Average pooling, S = 2	
		Conv 1 **×** 1, C = 192	
	MedViT block	$\left[\begin{array}{c} {ECB}^{192}\;\times \;3\\ {LTB}^{256}\;\times \;1 \end{array}\right]\times 1$	
Stage 3	Patch embedding	Average pooling, S = 2	
		Conv 1 **×** 1, C = 384	
	MedViT block	$\left[\begin{array}{c} {ECB}^{384}\;\times \;4\\ {LTB}^{512}\;\times \;1 \end{array}\right]\times 2$	$\left[\begin{array}{c} {ECB}^{384}\;\times \;4\\ {LTB}^{512}\;\times \;1 \end{array}\right]\times 1$
Stage 4	Patch embedding	Average pooling, S = 2	
		Conv 1 **×** 1, C = 768	
	MedViT block	$\left[\begin{array}{c} {ECB}^{768}\;\times \;2\\ {LTB}^{1024}\;\times \;1 \end{array}\right]\times 1$	
Path dropout		0.1	0.05
Number of parameters (M)		31.14	24.50

In the above table, C and S represent the number of channels and stride in convolutional layers, respectively. Also, the superscript of the ECB and LTB blocks indicates the output dimension.

### Stitching MedViTs

**Fig 3:**
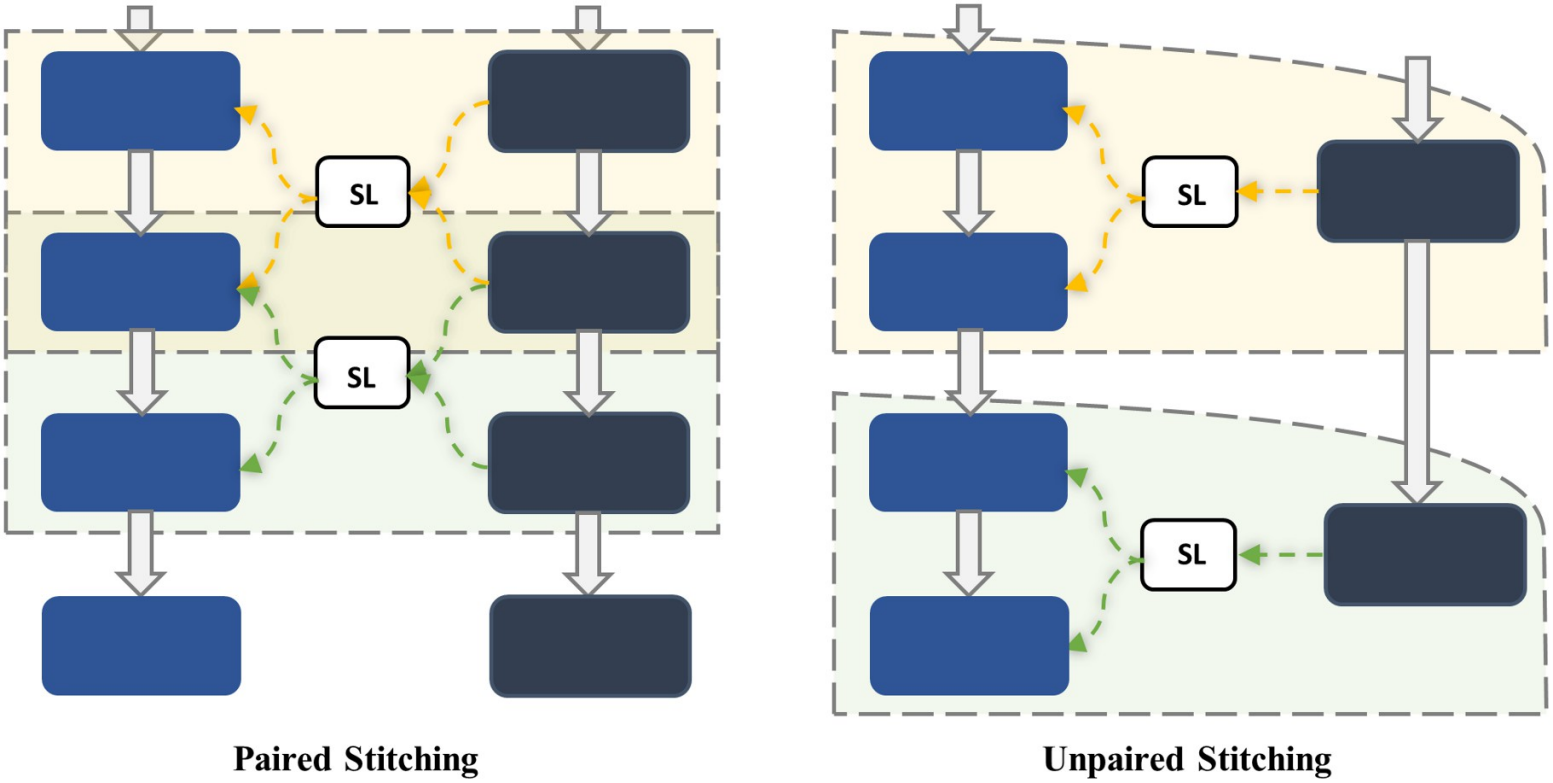
Paired stitching and unpaired stitching strategies. This visualization demonstrates how paired and unpaired stitching strategies select appropriate layers for stitching with anchors of the same length and different lengths, respectively.

Pan et al. in [26] proposed a low-cost neural network architecture search method to find an optimal architecture in efficiency-accuracy trade-off by having two or more anchors. A point to mention in choosing layers for stitching is paired and unpaired stitching strategies, which in the proposed method are used to stitch stages of the same length and different lengths, respectively. As shown in Fig 3, in the paired strategy, a moving window moves on the models with step *s* = 1 and length *k* = 2 and extracts different configurations for searching. Furthermore, in the unpaired strategy, the anchor with a smaller length is placed as a criterion, and each layer is proportional to the length of the larger anchor, with more than one of the larger anchor layers forming a candidate for the stitching layer (SL).

According to the proposed algorithm, between each pair of configurations extracted as the *m*-th layer of the (*i*+1)-th anchor, the *n*-th layer of the *i*-th anchor, a linear network (stitch) is defined to transform the activation maps of the *n*-th layer of the *i*-th anchor to activation maps of the (*m*−1)-th layer of the (*i*+1)-th anchor. Next, on a mini-batch, each stitch is initialized based on the closed-form response to find the transformation with the least square error. The algorithm iteratively selects stitches and updates their parameters using the stochastic gradient descent (SGD) optimization algorithm. This combination of the proposed initialization and SGD optimization facilitates smoother convergence during stitching, outperforming Kaiming initialization on average within a few epochs (specifically, fewer than 10 epochs for each candidate model) [26]. In other words, if we denote *l* as the number of defined stitch layers, the stitching process typically requires approximately *l* times the number of epochs needed for fine-tuning a single model. Notably, each stitch is implemented as a single-layer convolutional network with a 1×1 kernel size, chosen for simplicity and minimal impact on the inference time of the final model.

In this study, we stitched only two anchors in one direction (smaller to larger model) in each search. Considering the different lengths of the anchors, we followed the unpaired strategy for stitching. Also, considering the similarity between consecutive configurations, we kept only one of the two successive candidate configurations to reduce the search space. Following the default settings in [26], we set a batch size of 100 for initializing stitch layers. It is also worth mentioning that despite the suggested method in [26], we did not utilize a teacher model or distillation-based stitching for simplicity and due to a lack of open-source out-of-the-shelf models for AMD or eye disease detection based on OCT images. We have provided high-level pseudocodes for our pre-training and stitching methods in Algorithm 1 and 2, respectively.

**Algorithm 1**. **Pre-train MedViT.**

Inputs: *model type*, *dataset*

1: Split the given dataset to train and validation sets with a ratio of 80 to 20, respectively.

2: *best area under curve* = 0

3: **for** *i*_*epoch*_ in range (*number of epochs for pre-training*) **do**:

4: **for** *i*_*step*_ in range (*number of batches in the training set*) **do**:

5:  Get the next batch of data.

6:  Execute a training step

7: **end for**

8: *validation area under curve* = Calculate the average of the area under the receiver operating characteristic curve of the model on the validation set

9: **if** *validation area under curve* > = *best area under curve* **do**:

10:  *best model* = Save current checkpoint

11:  *best area under curve* = *validation area under curve*

12: **end if**

13: **end for**

14: **return** *best model*

**Algorithm 2**. **Stitching MedViTs for five-fold cross-validation.**

Inputs: *source dataset*, *target dataset*

1: Split the *target dataset* into 5 folds.

2: **if** *source dataset* **!=** *target dataset* **do**:

3: *anchor 1* = Pre-train MedViT (Micro, *source dataset*).

4: *anchor 2* = Pre-train MedViT (Tiny, *source dataset*).

5: **end if**

6: **for** *i*_*fold*_ in range (5) **do**:

7: *test set* = Load the *i*-th fold.

8: *remained set* = Subtract *test set* from *target dataset*.

9: **if** *source dataset* **= =** *target dataset* **do**:

10:  *anchor 1* = Pre-train MedViT (Micro, *remained set*).

11:  *anchor 2* = Pre-train MedViT (Tiny, *remained set*).

12: **end if**

13: *configurations* = Given anchors, get all possible configurations based on the unpaired stitching strategy and remove those with odd indexes.

14: **for** *config* in *configurations* **do**:

15:  Initialize the *config*’s stitch layer based on the least-square matching response for a batch size of 100 samples from the *remained set*.

16: **end for**

17: **for** *i*_*iteration*_ in range (*number of iterations for stitching*) **do**:

18:  Get the next batch of data.

19:  Select a random configuration

20:  Execute a training step based on the selected configuration.

21: **end for**

## Experiments and results

**Fig 4:**
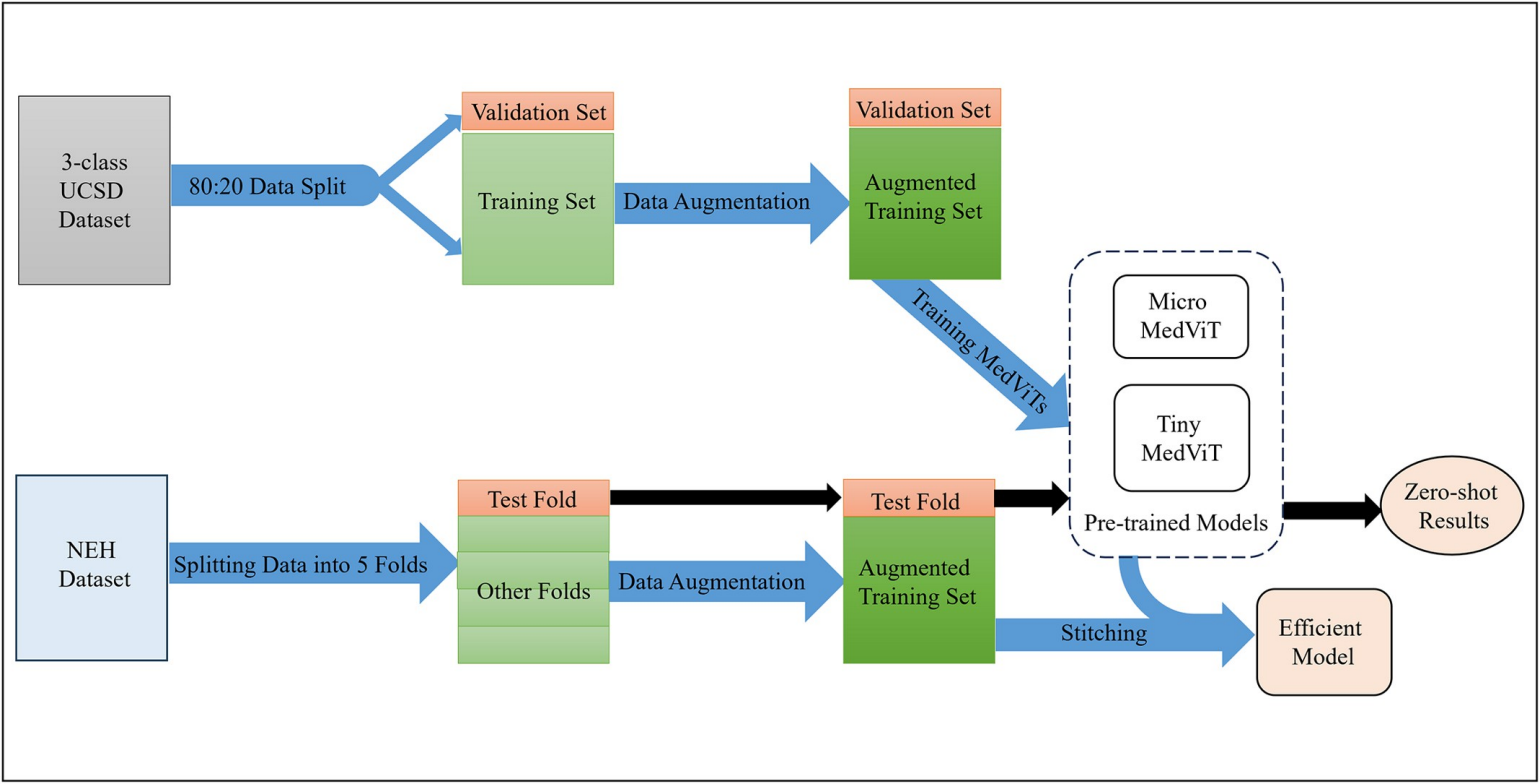
Utilizing stitchable neural networks as an architecture search method based on pre-trained MedViTs on UCSD. Utilizing stitchable neural networks results in an efficient model for classifying retinal OCT images from the NEH dataset.

**Fig 5:**
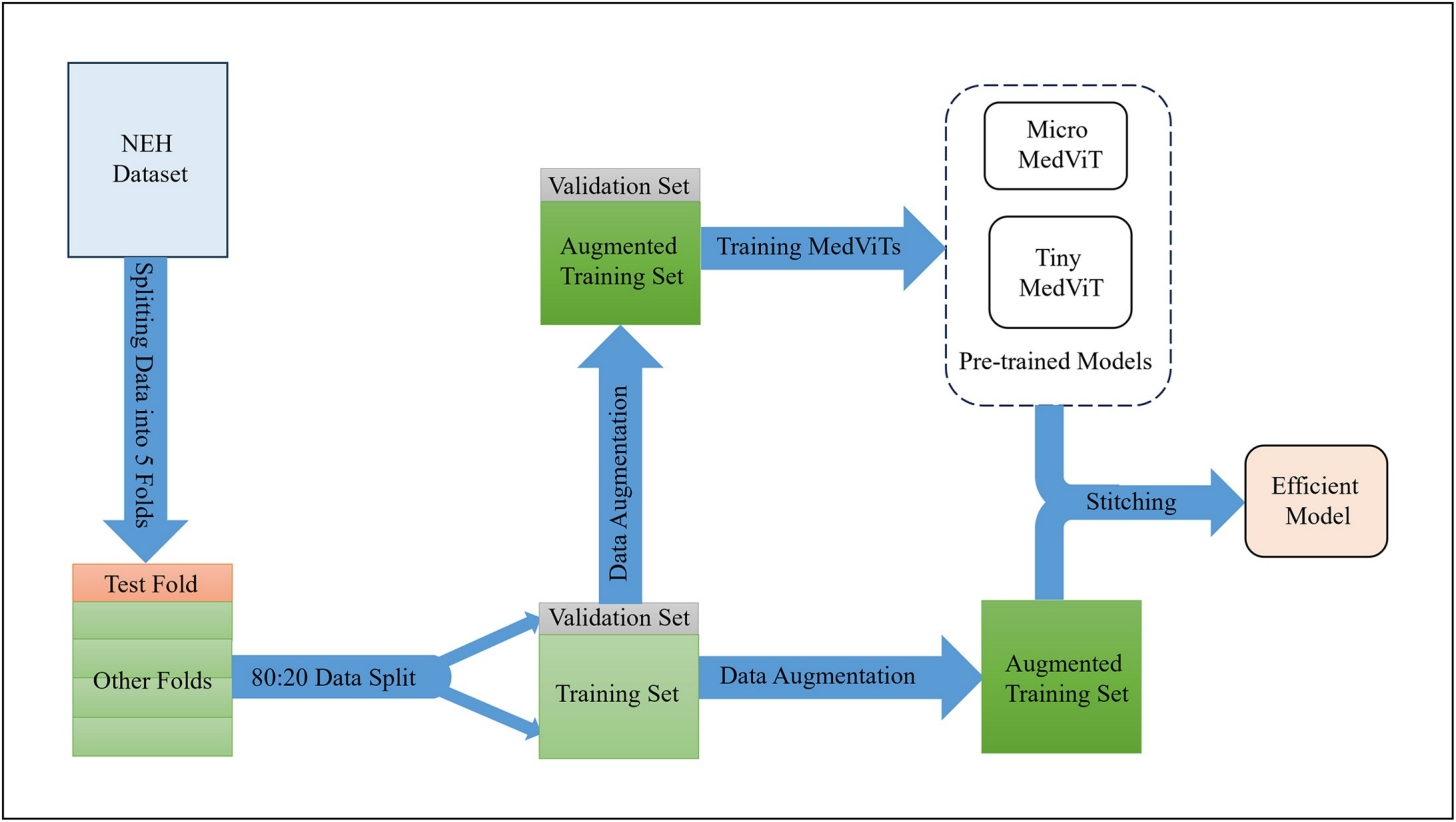
Utilizing stitchable neural networks as an architecture search method based on pre-trained MedViTs on NEH. Utilizing stitchable neural networks results in a model with superior performance compared to the state-of-the-art model for classifying retinal OCT images from the NEH dataset.

This section describes the details of the experiments and compares the results with previous studies. Figs 4 and 5 demonstrates the overall flowchart of the experiments in in-distribution and out-of-distribution stitching scenarios, respectively.

### Datasets

In this study, we used OCT images from publicly available datasets of the University of California San Diego [29] and Noor Eye Hospital [28].

#### NEH-v2 dataset

To evaluate the method presented in this study, we used five-fold cross-validation on the second version of the NEH dataset. This dataset consists of 16,822 B-scan OCT images with normal, drusen, and CNV labels, which were imaged from the eyes of 441 people over 50 years old and without any other retinal complications by the Heidelberg SD-OCT imaging system at Noor Eye Hospital in Tehran, Iran.

All images have an image quality of Q≥20 (measured by the imaging system) and have been labeled by specialists and saved in dimensions (768,496) [41]. We only kept 12649 images with the worst labels for each patient and discarded the rest to compare our results with previous studies. The entire dataset is randomly divided into five parts to use five-fold cross-validation. At each stage, one part is used as test data, and the rest is divided into training and validation data with a ratio of 80 to 20, which is used to train the models and fine-tune their hyperparameters.

#### UCSD dataset

**Fig 6:**
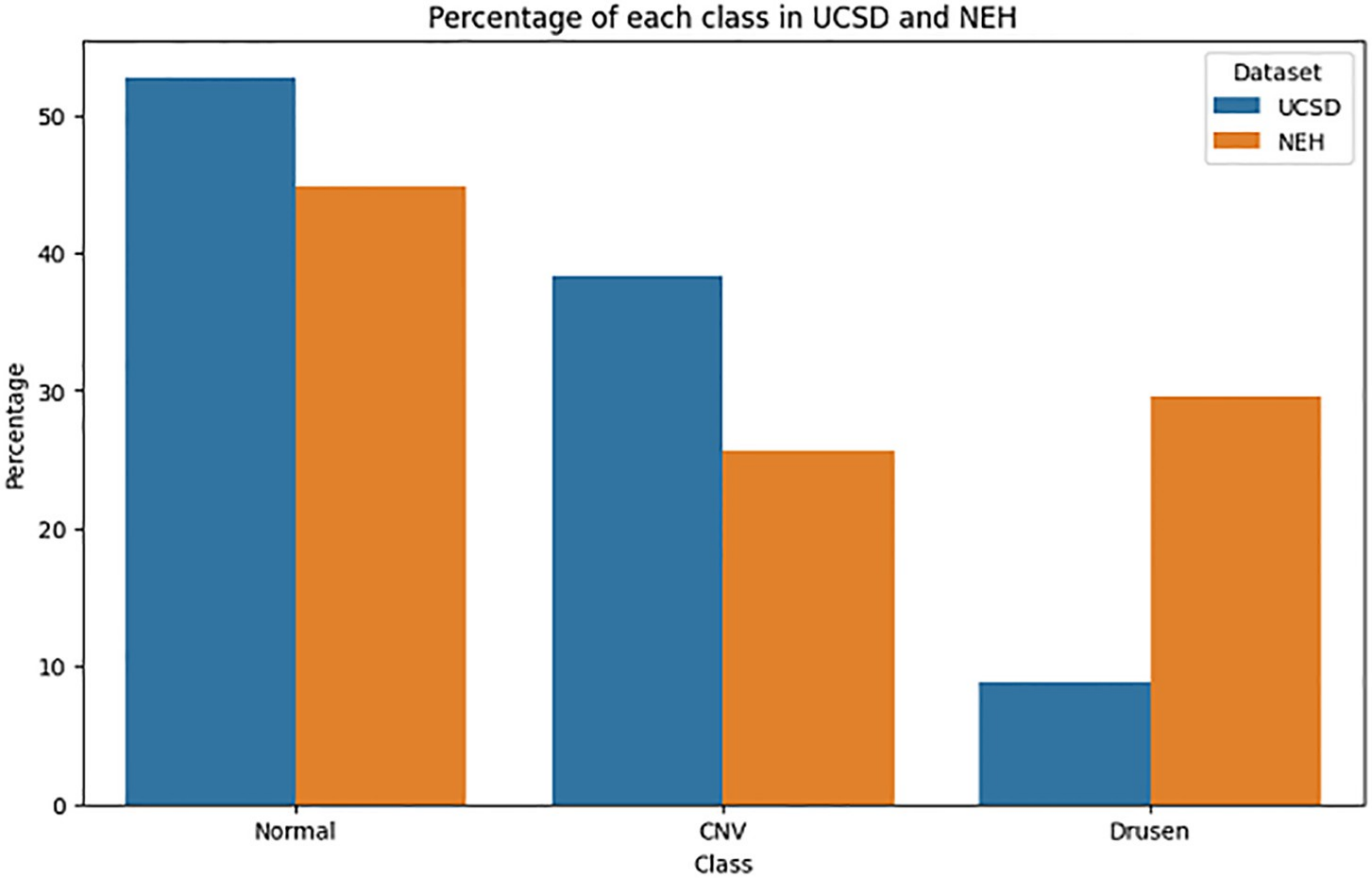
Class distribution percentages within the NEH and UCSD datasets.

The UCSD dataset contains 108,312 B-scan OCT images from the eyes of 4686 people in different dimensions, placed in 4 classes labeled drusen, normal, CNV, and DME [14]. In order to match the NEH target dataset, we used only 96961 images of drusen, normal, and CNV classes and divided them into train and validation parts with a ratio of 80 to 20. We tested the pre-trained models on this dataset in a zero-shot manner using the images of the NEH dataset. Fig 6 shows the class distribution of utilized images from the UCSD and NEH datasets.

#### Data augmentation

To handle the different sizes of images from the UCSD and NEH datasets and reduce the computational cost, we resized all the images in the dataset to (224,224) dimensions, which was the only preprocessing performed. For data augmentation, the training data used in each step of the five-fold cross-validation flow through of random affine, color jitter, and random horizontal flip transformations, in order. Also, at the end of this step, all images were converted to tensor and normalized. Table 4 shows the parameter values of each mentioned transformation.

**Table 4:** Transformations performed for data augmentation.

Augmentation type	Parameter	Value
Random affine	Rotation (degrees)	(-20, 20)
	Translate (relative)	(0.05, 0.2)
	Shear (degrees)	(-10, 10)
	Scale (relative)	(0.8, 1.2)
Color jitter	Brightness (relative)	(0.8, 1.2)
	Contrast (relative)	(0.8, 1.2)
	Saturation (relative)	(0.8, 1.2)
Random horizontal flip	Probability	0.5
Normalization for samples from the NEH dataset	Mean	0.13
	Standard deviation	0.194
Normalization for samples from the UCSD dataset	Mean	0.19
	Standard deviation	0.215

### Experimental setup

To reach the final stitched model, first, the micro and tiny MedViT models are trained separately in 75 epochs on the UCSD and NEH datasets, resulting in pre-trained models. In the next step, we stitched the pre-trained models in 100 epochs on the target dataset, the NEH dataset. In both stages, for hyperparameter initialization, we have followed suggested values of previous studies [25,26]. we used the learning rate with the starting value of 5e-5, decayed by a factor of 0.1 in epoch 50 for pre-training models. For stitching, its value is scaled linearly with the batch size value. Furthermore, the learning rate was adjusted using a cosine scheduler as the training progressed. In all experiments, focal loss [30] was used as a loss function along with AdamW optimizer due to the dataset imbalance. Focal loss is a generalized version of cross entropy loss that imposes a higher penalty on the model when incorrectly labels complex dataset samples. We have provided pre-training and stitching hyperparameters in Table 5.

**Table 5:** Pre-training and stitching hyperparameters.

Hyperparameter	Value in pre-training	Value in stitching
Learning rate	5e-5	5e-5
Batch size	32	32
Learning rate scheduler	Step scheduler	Cosine annealing scheduler
Decay factor	0.1	-
Milestones	50	-
Optimizer	AdamW	AdamW
Loss function	Focal loss	Focal loss

## Results

To better evaluate the presented method and compare the results with previous studies, we will calculate and show the measures of accuracy, sensitivity and specificity and each studied model’s number of parameters.

### Pre-trained models

In this part, we will present the results of the pre-trained models on the UCSD and NEH datasets in the classification of NEH dataset images. According to our knowledge, zero-shot results have yet to be done on retinal OCT datasets for the classification task, so researchers in this field can use the developments of this section to conduct additional research and improve the results of models in the out-of-distribution mode. Table 6 shows the results of the micro and tiny MedViT models. Table 6 demonstrates that the zero-shot outcomes of the tiny model are better than the micro model. In contrast, in the in-distribution mode, the two tiny and micro models have similar results, while the number of parameters of the micro model is less.

**Table 6:** Results of the pre-trained micro and tiny MedViT models on the NEH dataset.

Model	Specificity	Sensitivity	Accuracy	Params(M)	Source dataset
micro MedViT	0.928 ±0.002	0.808 ±0.007	0.828 ±0.007	24.5	**UCSD**
tiny MedViT	0.933 ±0.002	0.824 ±0.007	0.841 ±0.007	31.1	**UCSD**
micro MedViT	0.987 ±0.001	0.975 ±0.002	0.977 ±0.002	24.5	**NEH**
tiny MedViT	0.986 ±0.002	0.974 ±0.004	0.977 ±0.004	31.1	**NEH**

### Stitched MedViT

In this section, we will compare the average results of the five-fold cross-validation of the final models obtained from stitching the pre-trained models introduced in the previous section, along with former studies on the NEH dataset. Table 7 shows the results of the proposed method and earlier studies in terms of specificity, sensitivity, accuracy, and number of parameters and their fluctuations based on the confidence level of 95%. According to the results, the proposed stitched MedViT found two competitive models with average accuracies of 94.8% and 95.0% among the candidate models in the search space, with 30.5 and 31.1 million parameters, respectively. Although the final best model achieved 2.9% higher average accuracy when we initially trained anchors on the NEH dataset, it should be noted that in another scenario where we train the anchors on the UCSD dataset, it has more practical relevance and is closer to real-world situations, which is because pre-trained models are often trained on datasets other than the target dataset. Our out-of-distribution stitched MedViTs achieved comparable results with state-of-the-art models in terms of accuracy. Also, our in-distribution stitched MedViTs were able to outperform state-of-the-art models in [44] by a margin of at least 2.8% in terms of accuracy, specificity, and sensitivity. While previous models have notable gaps between their specificity and sensitivity measurements, our proposed models exhibit more balanced specificity and sensitivity, which gives the user more confidence in both true negative and true positive samples, respectively. It is important to acknowledge that, unlike our training and stitching strategies, all previous studies in this comparison did not incorporate any data augmentation techniques during their training process. It is also worth mentioning that, in exchange for the increase in accuracy achieved by the final model, due to the higher number of parameters in the anchors, which belong to the MedViT family, the number of parameters in the final model is more significant than in previous studies, increasing the model’s size. Among the prior studies, InterNRL stands out for its interpretability, providing users with insights into the patterns influencing the classification of OCT images. In comparison, stitched MedViTs demonstrate reduced interpretability. However, it is possible to investigate challenging decisions in MedViT using class activation maps as shown in [25].

**Table 7:** Comparing the five-fold cross-validation results of the presented method with previous studies on the NEH dataset.

Method	Specificity	Sensitivity	Accuracy	Params(M)	Notes
Multi-size Kernels (MSK*ξ*MP) [8]	0.943	0.885	0.892	-	**-**
InterNRL [32]	0.959	0.910	0.919	-	**-**
Pruned FPN-VGG16 [44]	0.953	0.91	0.946	15.7	**-**
BiFPN-VGG16 [44]	0.954	0.914	0.948	16.2	**-**
PAN-VGG16 [44]	0.957	0.921	0.950	15.9	**-**
Micro MedViT [this study]	0.987 ±0.001	0.975 ±0.002	0.977 ±0.002	24.5	**-**
Stitched MedViTs (config_id = 9) [this study]	0.969 ±0.002	0.942 ±0.005	0.948 ±0.004	30.5	**Anchors pre-trained on UCSD**
Stitched MedViTs (config_id = 7) [this study]	0.971 ±0.002	0.945 ±0.004	0.950 ±0.004	31.1	**Anchors pre-trained on UCSD**
Stitched MedViTs (config_id = 9) [this study]	0.986 ±0.002	0.975 ±0.003	0.978 ±0.003	31.0	**Anchors pre-trained on NEH**
Stitched MedViTs (config_id = 6) [this study]	0.988 ±0.002	0.976 ±0.003	0.979 ±0.002	31.1	**Anchors pre-trained on NEH**

## Conclusion and future work

In this study, we aimed to achieve an optimal trade-off between efficiency and accuracy in classifying retinal OCT images into three classes: drusen, CNV, and normal, using the pre-trained MedViT models, which are hybrid CNN-transformer networks. To achieve this, we introduced the “micro” model to the MedViT family and demonstrated that despite having fewer parameters, the added model performs similarly to the “tiny” model. Furthermore, we evaluated the “tiny” and “micro” models in a zero-shot setting and through five-fold cross-validation on the NEH dataset. We used them as anchors for the stitching process. As expected, we observed that stitching pre-trained models on the target dataset results in a better classifier than out-of-distribution stitching in a constant number of epochs. The findings of this study provide empirical support for the applicability hypothesis of neural network stitching as an effective fine-tuning method. Specifically, this approach can lead to an optimal model in terms of both accuracy and architectural considerations for specific domain tasks, such as OCT image classification for AMD detection.

One overlooked aspect of this study is the difference in the number of classes between the source and target datasets. In practice, pre-trained models on the UCSD dataset are expected to distinguish between four classes, while for the target dataset, NEH, the model needs to have 3-output layers. To address this issue in a real-world scenario, adding a fine-tuning step on pre-trained models with a projection head containing 3-output layers before the stitching process is necessary.

Additionally, it is essential to consider the practical usability of the obtained model. Therefore, ongoing efforts should focus on reducing model errors and minimizing the number of parameters in the model. When using a large-scale model, even a 1% error in model accuracy can lead to significant cost and time losses when testing individuals. Furthermore, it is crucial to evaluate the models provided in the medical field in a zero-shot manner. This is because the input to the models may suffer from distribution shifts due to differences in the systems used for image acquisition and processing. In such cases, zero-shot results will be closer to the models’ real-world performance. The other overlooked aspect of the proposed method pertains to the usability of large and domain-specific pre-trained models as input models for stitching. Theoretically, employing large models pre-trained on diverse datasets as anchors in stitching could yield improved results. However, these large models predominantly learn representations of OCT images in unsupervised or self-supervised learning paradigms due to limited labeled data availability and the utilization of varied datasets with distinct labeling strategies. A potential avenue for future research involves extending the stitching method to representation learning scenarios on target datasets using large pre-trained models.

Age-related macular degeneration is one of the most significant factors contributing to vision loss in elderly individuals, especially in developed countries. With the increase in the aging population worldwide, there is a need for automated and rapid methods for early AMD detection and monitoring. One non-invasive method that can be employed for this purpose is optical coherence tomography images. OCT images allow physicians to diagnose and track treatment effects in patients. Unlike manual staging systems, automated AMD staging systems remain unaffected by fatigue or mental state and can yield rapid results in large-scale visual health monitoring of elderly individuals. Therefore, the development of intelligent systems for the classification and segmentation of OCT images is of utmost importance for preventing the progression of AMD and aiding in its treatment. Stitchable neural networks have the potential to assist developers of automatic AMD detection systems in achieving fast and accurate model deployment by leveraging the knowledge of established state-of-the-art deep neural networks.

## Supporting information

S1 Graphical abstract (JPG)
