# Supplementary figures and images for: Stitched vision transformer for age-related macular degeneration detection using retinal optical coherence tomography images
Source: PLoS One. 2024 Jun 5;19(6):e0304943. doi: 10.1371/journal.pone.0304943 (PMC11152295; doi:10.1371/journal.pone.0304943)

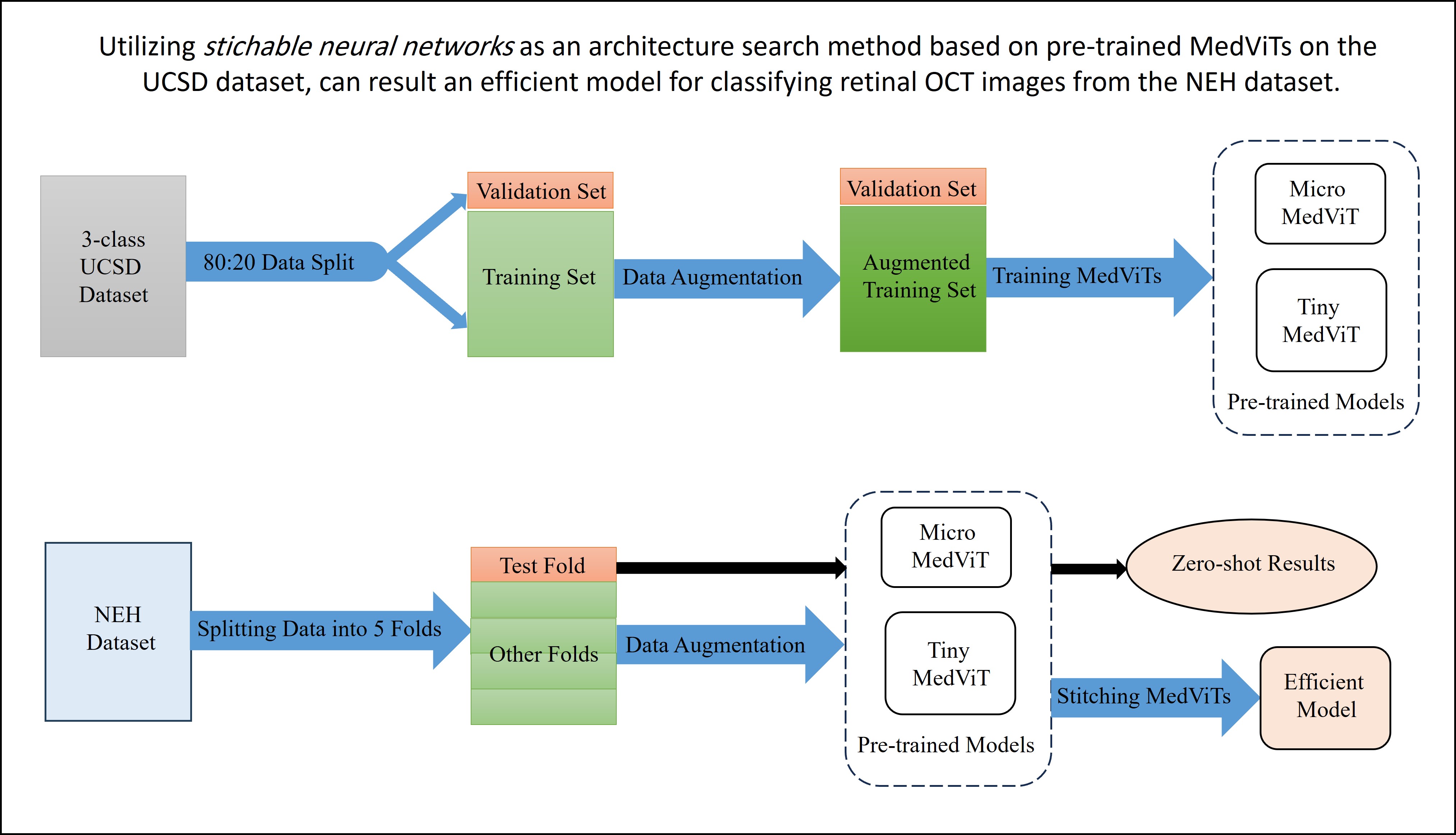

Supplement: S1 Graphical abstract — (JPG) [file pone.0304943.s001.jpg]
